# D-Aspartic Acid in Vertebrate Reproduction: Animal Models and Experimental Designs ‡

**DOI:** 10.3390/biom9090445

**Published:** 2019-09-03

**Authors:** Maria Maddalena Di Fiore, Raffaele Boni, Alessandra Santillo, Sara Falvo, Alessandra Gallo, Sabrina Esposito, Gabriella Chieffi Baccari

**Affiliations:** 1 Dipartimento di Scienze e Tecnologie Ambientali, Biologiche e Farmaceutiche, Università della Campania L. Vanvitelli, Via Vivaldi 43, 81100 Caserta, Italy; 2 Dipartimento di Scienze, Università della Basilicata, Via dell’Ateneo Lucano 10, 85100 Potenza, Italy; 3 Dipartimento di Biologia ed Evoluzione degli Organismi Marini, Stazione Zoologica Anton Dohrn, Villa Comunale, 80121 Napoli, Italy

**Keywords:** D-aspartate, N-methyl-D-aspartate, sex steroid hormones, steroidogenesis, spermatogenesis, testosterone, hypothalamus-pituitary-gonad axis

## Abstract

This article reviews the animal models and experimental designs that have been used during the past twenty years to demonstrate the prominent role played by D-aspartate (D-Asp) in the reproduction of vertebrates, from amphibians to humans. We have tabulated the findings of in vivo and in vitro experiments that demonstrate the effects of D-Asp uptake on hormone production and gametogenesis in vertebrate animal models. The contribution of each animal model to the existing knowledge on the role of D-Asp in reproductive processes has been discussed. A critical analysis of experimental designs has also been carried out. Experiments performed on wild animal species suggest a role of D-Asp in the mechanisms that regulate the reproductive cycle. Several in vivo and in vitro studies carried out on mouse and rat models have facilitated an understanding of the molecular pathways activated by D-Asp in both steroidogenesis and spermatogenesis, with particular emphasis on testosterone biosynthesis. Some attempts using D-Asp for the improvement of reproductive activity in animals of commercial interest have yielded mixed results. The increased transcriptome activity of enzymes and receptors involved in the reproductive activity in D-Asp-treated broiler roosters revealed further details on the mechanism of action of D-Asp on the reproductive processes. The close relationship between D-Asp and reproductive activity has emerged, particularly in relation to its effects exerted on semen quality, proposing therapeutic applications of this amino acid in andrology and in medically-assisted procreation techniques.

## 1. Introduction

During the last two decades, an increasing volume of evidence has shown the occurrence of D-aspartate (D-Asp) in the endocrine and neuroendocrine tissues of vertebrates and its central role in the regulation of reproductive activity [1,2,3,4,5]. The investigations have focused on the major vertebrate classes and have demonstrated that D-Asp is involved in several steps of steroidogenesis, all of which follow one another to regulate the synthesis and/or release of sex-steroid hormones.

The mechanisms of origin, turnover, and metabolism of free D-Asp in vertebrate tissues remain to be established. D-Asp may be produced by the degradation of proteins that are derived from the diet or obtained from intestinal bacteria containing D-Asp. D-Asp is widely present in several plants [6], due to which, some foods that are particularly rich in D-Asp might be useful in conditioning the reproductive activity of animals. Furthermore, animal tissues appear to contain the complete molecular machinery required to regulate D-Asp homeostasis, as they can synthesize, release, take up, and degrade this amino acid. Wolosker et al. [7] suggested that D-Asp is synthesized from L-Asp, thus supporting the existence of the enzyme aspartate racemase, a strong candidate for D-Asp synthesis in mammals. D-Asp is essentially metabolized by the D-Asp oxidase (D-AspO), a peroxisomal flavoprotein that specifically metabolizes D-Asp into oxaloacetate, NH_3_, and H_2_O_2_ [8]. In Ddo ^-/-^ mice, the concentration of D-Asp was twice of that in the wild type [9].

Particular attention has been paid by researchers to studying the effects of D-Asp on endocrine activity. This is due to the high concentrations of D-Asp found in the endocrine glands showing an elective form of accumulation following D-Asp administration. The effects of D-Asp and its methylated form N-methyl-D-aspartate (NMDA) on the production of sex hormones are well-known and conserved across the various species examined. In contrast, the studies on the other hormones yielded conflicting results [2]. In mammals, the most actively pursued research on the effects of D-Asp and NMDA in reproductive mechanisms in males involves penile erection, ejaculation, sexual potency, and libido, as well as their direct effects on semen quality. However, the available information on these issues is scarce, fragmented, and the knowledge needs to be expanded.

Although the receptors of D-Asp remain to be characterized, the postsynaptic response of D-Asp has been well-studied and several L-glutamate receptors (GluRs), such as NMDA receptors (NMDAR), are known to respond to D-Asp. These receptors may be mainly grouped as ionotropic (containing an ionic channel) and metabotropic (acting via G-protein coupling) types [10]. L-glutamate receptors also include the ionotropic α-amino-3-hydroxy-5-methyl-4-isoxazole propionic acid receptor (AMPAR). Recent studies have demonstrated that both NMDAR and AMPAR are expressed in the testes of rat and mouse and are particularly evident in spermatogonia and Leydig cells [11,12,13,14].

Herein, we review all the animal models and experimental designs that have been used during the past 20 years to demonstrate the prominent role played by D-Asp in the reproduction of all vertebrates from amphibians to humans. We have tabulated the in vivo and in vitro experiments, as well as the effects induced by the D-Asp uptake on hormone production and gametogenesis in vertebrate animal models, categorized into the wild, laboratory, and livestock animals. Separate sections are dedicated to primates and humans. The contribution of each animal model to the existing knowledge on the role of D-Asp in reproductive processes has also been discussed. A critical analysis of experimental designs has also been carried out.

## 2. Wild Animals

The most commonly used animal models in the initial studies on the role of D-Asp in reproduction were the edible frog (*Pelophylax esculentus*) and the Italian wall lizard (*Podarcis sicula sicula*). These are seasonally reproductive species with a physiological on/off switch for the gonadic activity. Owing to this property, they are widely studied in the field of comparative endocrinology [15,16,17,18,19,20,21,22,23,24]. The breeding of these species occurs only during the spring season. Early studies have revealed that the gonadic levels of D-Asp were directly linked with the cyclic levels of sex hormones, suggesting the involvement of this amino acid in the regulation of seasonal reproductive activity. The concentrations of D-Asp in both testis and serum were positively correlated with the testosterone (T) levels, showing its highest levels in sexually active animals [25,26,27,28,29]. In contrast, the ovaries of *P. esculentus* and *P. s. sicula* showed a reverse correlation between the levels of D-Asp and T. High T concentrations in the ovaries and blood plasma of reproductive females coincided with the lowest concentration of D-Asp in the ovaries. Conversely, low T concentrations in the ovaries and plasma of post-reproductive females coincided with the highest levels of D-Asp in the ovaries [30,31,32]. A confirmation of the relationship between D-Asp and T biosynthesis was found using in vitro studies with a wild bird model, such as a mallard (*Anas platyrhynchos*) [25].

### 2.1. In Vivo Experiments

As shown in Table 1a (long-term experiments), D-Asp that was administered intraperitoneally accumulated in *P. esculentus* testes and brain, where it activated the estrogen and androgenic pathways in the reproductive and non-reproductive phases, respectively. An increase in the level of steroidogenic acute regulatory protein (StAR) transcripts, which encoded the regulatory enzyme carrying cholesterol from the outer to inner mitochondrial membrane, was observed in the testes of both reproductive and non-reproductive animals treated with D-Asp. Furthermore, the administration of D-Asp in male frogs during the breeding season induced an increase in the levels of P450 aromatase (P450 aro, a key enzyme in the estrogen synthetic pathway, converting T into 17β-estradiol) transcripts, as well as a strong increase in 17β-estradiol (E2) levels in the testes (Table 1a) [33]. In the brain, higher levels of P-CREB, E2, and estrogen receptor-α (ERα) mRNA, as well as lower levels of T and androgen receptor (AR) mRNA, were described (Table 1a) [34,35].

In the testes of *P. esculentus* during the non-breeding season, treatment with D-Asp enhanced the levels of StAR and 5α-reductase (5α-Red), the enzyme converting T in dihydrotestosterone, (DHT) mRNAs, as well as T, but decreased the levels of E2 (Table 1a) [33]. Moreover, a higher concentration of D-AspO was also described. Finally, the increased concentration of D-Asp in the brain induced the local increase of both D-AspO activity and levels of caspase-3 protein [36].

In vivo short-term experiments (Table 1b) involving a single intraperitoneal injection of D-Asp demonstrated the significant accumulation of D-Asp in the gonads of *P. esculentus* [26,31,37] and *P. s. sicula* [27,30]. The higher rate of D-Asp uptake in the gonads following a single D-Asp administration (Table 1b) may be explained by an increased activity of D-AspO in animals subjected to long-term administration of the amino acid [36].

Outside the breeding season, *P. esculentus* showed a significant increase in the levels of both testicular and serum T three hours after D-Asp administration (Table 1b) [26,29]. In contrast, in either breeding or non-breeding female frogs, the accumulation of exogenous D-Asp decreased the levels of ovarian T (Table 1b) [29,31].

Exogenous D-Asp accumulates at a higher rate in the testis of non-reproductive *P.s. sicula* compared to the reproductive ones [27,38,39]. This probably explains the greater increase in the levels of testicular and serum T that was observed in the out-of-breeding-season animals treated with D-Asp [27]. An increase in the levels of c-kit, tyrosine kinase activity, and proliferating cell nuclear antigen (PCNA) was also observed 3–6 h following D-Asp administration. These findings were confirmed using immunohistochemical studies [28]. On the contrary, the administration of this amino acid significantly decreased the levels of E2 in both serum and testis of the breeding and out-of-breeding season males (Table 1b) [27].

The elevated uptake of exogenous D-Asp in female *P. s. sicula* outside the breeding season caused a decrease in the T levels, and conversely, a considerable increase of E2 levels in both ovary and blood at 3–6 h after treatment (Table 1b) [30]. The levels of D-Asp and sex hormones progressively reached baseline values within 24 h after the treatment.

### 2.2. In Vitro Experiments

The in vitro experiments, performed using isolated ovarian follicles, confirmed the results of in vivo experiments that were carried out on *P. esculentus* females during the breeding season [31]. Subsequent to the supplementation of D-Asp in the culture medium, a lower percentage of T levels produced by ovarian follicles was observed in the absence or presence of pituitary tissue (Table 2). Nevertheless, a significant increase in the activity of aromatase was observed in the ovarian tissue or ovarian follicle of *P. s. sicula* after D-Asp addition into the culture medium (Table 2) [30].

Similarly, when slices of the testes of *A. platyrhynchos* were incubated in a medium containing D-Asp, a significant dose-dependent increase in the levels of T was observed in the culture medium (Table 2) [25].

In summary, the in vivo experiments conducted on wild vertebrate species have suggested a role of D-Asp in mechanisms regulating the reproductive cycle. D-Asp administration has been shown to promote the activation of androgenic pathway and to inhibit the estrogenic pathway in male lizards in both reproductive and non-reproductive periods. In the female frogs, a decrease of T, and vice versa, an increase of E2, was observed. These findings were confirmed by the in vitro experiments indicating a direct effect of D-Asp on either ovarian aromatase activity or testicular T production. Some contrasting effects induced by D-Asp in testes and ovaries may be ascribed to transient effects of this amino acid, as demonstrated in short-term experiments.

## 3. Laboratory Animals

In the brain, the hypothalamus shows the highest abundance of D-Asp in the entire nervous system. Among the endocrine glands, the highest D-Asp concentration is found in the adenohypophysis [41,42]. In the pineal and adrenal glands, the concentration of D-Asp shows a temporal increase at certain stages. In the pineal gland, D-Asp concentration changes in relation to the circadian biorhythm and acts as a negative regulator of melatonin synthesis [43]. In addition, D-Asp levels increase in the testis just before birth and during sexual maturation. These distributional patterns and developmental changes may be attributed to differences in the abilities of various tissues to dispose of D-Asp or may reflect alterations according to specific functional requirements of D-Asp [44].

The localization of D-Asp within the testes was tracked in rats and analyzed using enzymatic and chromatographic methods [45], as well as by immunohistochemical and biochemical techniques [46]. The highest concentrations of D-Asp were found in testicular venous blood plasma, with slightly lower concentrations in rete testis fluid and epididymal spermatozoa. Lower levels were found in testicular parenchymal cells, luminal fluid from the seminiferous tubules, and interstitial extracellular fluid. Nevertheless, all these values were still higher than those found in the peripheral blood plasma [45]. Subcellular localization of D-Asp in the components of the testes revealed its immunoreactivity in the cytoplasm of Leydig and germ cells, especially around regions that were rich in elongated spermatids. D-Asp was also localized in the cytoplasm of spermatocytes, whereas it was found in negligible amounts in the Sertoli cells [46].

Several reports have shown that administration of D-Asp exerts potent positive effects on the reproduction of laboratory animals by influencing the synthesis and release of gonadotropin, gonadic steroid production, and spermatogenesis [2,47].

### 3.1. In Vivo Experiments

In Table 3, we have reported the effects of chronic administration of D-Asp (long-term experiments) on mice and rats. Sexually immature (seven-week-old) male mice C57BL/6NT (B6N) were treated with a dose of 20 mM D-Asp in drinking water [48]. Parameters such as efficiency of in vitro fertilization (IVF), the quality of frozen-thawed sperm, as well as luteinizing hormone (LH), epitestosterone, and T levels were investigated. After two and four weeks of treatment, the IVF rate and the total sperm motility, as well as LH, epitestosterone, and T levels in the serum and testes, were significantly higher in the D-Asp-treated males (Table 3) [48].

Chandrashekar and Muralidhara [49] demonstrated that the chronic administration of massive doses of D-Asp induced a widespread oxidative stress in the testes of prepubertal Wistar rats (Table 3). D-Asp treatment significantly increased the levels of reactive oxygen species (ROS) in both cytosol and mitochondria. Increased levels of malondialdehyde (MDA), lipid peroxides (LPO), hydroperoxides (HP), and nitric oxide (NO) in the testes were accompanied by enhanced glutathione levels and elevated activities of the glutathione-dependent enzymes catalase, D-AspO, and 3β-hydroxysteroid dehydrogenase (Table 3).

A study on sexually mature Wistar rats demonstrated that the long-term oral administration of D-Asp induced significant uptake of the amino acid in the testes, pituitary gland, serum, and brain. An increase in T and androstenedione levels in the serum and testes was demonstrated, together with the increased serum LH levels (Table 3) [40,50]. The levels of T were still higher (1.27-fold) than the control, even after three days following the suspension of the treatment, whereas at this time the levels of LH were back at baseline. One plausible explanation for this observation was that significant amounts of D-Asp remained accumulated in the testes for up to three days after the treatment suspension [50]. Furthermore, increased levels of StAR (mRNA and protein), P450 cholesterol side-chain cleavage (P450scc), 3β-hydroxysteroid dehydrogenase (3β-HSD), NR1 and NR2A (NMDAR subunits) mRNA, and AR protein were observed in the testes of D-Asp-treated rats, while the levels of P450 aro and ERα protein were decreased (Table 3) [13,14,15,16,17,18,19,20,21,22,23,24,25,26,27,28,29,30,31,32,33,34,35,36,37,38,39,40,41,42,43,44,45,46,47,48,49,50].

A chronic (30-day) treatment with D-Asp also affected sex steroid synthesis in rat brain, resulting in an increase in the levels of progesterone (P), T, and E2 in the brain (Table 3), which suggests a role played by this amino acid on the upregulation of the activity of steroidogenic enzymes in the nervous system [51]. This hypothesis has also been confirmed by in vitro experiments [51].

By using toxicants to induce the selective destruction of Leydig cells, such as methoxyacetic acid, a significant decrease in the D-Asp levels and a drop in T was observed, which resulted in the consequent modulation of spermatogenesis [46] (data not reported in the table).

Following short-term experiments in sexually mature male Wistar rats, a significant increase in the D-Asp levels occurred in the testes, adenohypophysis, hypothalamus, and adrenal glands within 1–8 h after the treatment [41,42], while in the epididymis, peak values were observed within 30 min [52] (Table 4). Among the endocrine glands, the adenohypophysis demonstrated the highest ability to accumulate the administrated D-Asp [41] and the serum levels of D-Asp peaked after 1 h following its injection (Table 4) [41]. D-Asp uptake induced a significant increase in NMDA in the testes, adenohypophysis, and hypothalamus within 1 h after D-Asp injection (Table 4). Compared to the other tissues, the hypothalamus showed the highest increase in NMDA levels [41]. These findings led to the hypothesis that the increase in NMDA in these tissues was due to the transformation of D-Asp into NMDA, which was supported by the expression of NMDA synthase, an *S*-adenosylmethionine-dependent enzyme, which could be observed in these tissues.

Consistent with this, microinjection of 0.20 nmol NMDA into the paraventricular nucleus (PVN) was observed to facilitate ejaculation during copulation by increasing the baseline lumbar splanchnic nerve activity (LSNA), and the dose of NMDA was positively correlated with the increased LSNA. Meanwhile, the level of norepinephrine in the plasma of rats that were injected with NMDA was considerably higher than that in the rats that were injected with saline. Thus, NMDAR in the PVN may facilitate ejaculation by enhancing the activity of the sympathetic system [53]. Furthermore, the administration of NMDA in male rats with specific lesions of LSt cells, a population of spinothalamic neurons, has demonstrated that glutamate provides a key afferent signal for ejaculation by activating the NMDA receptors in LSt cells [54].

A significant increase in the levels of P, T, LH, growth hormone (GH), and prolactin (PRL) in the serum was observed within 1–2 h after the administration of D-Asp, and after 5 h, these parameters reached the highest levels (Table 4) [41,42]. Intact and ovariectomized female rats that were injected intraperitoneally with NMDA [15–30 mg/kg body weight (b.w.)] and sacrificed at 0–45 min, showed increased levels of serum LH [55]. The highest levels of T and E2 were also observed in both the brain and epididymis within 5 h after the administration of D-Asp (Table 4). Furthermore, the injection of D-Asp induced an increase in the levels of DHT and the transcripts of 5αRed1/2, P450 aro, AR, and ERα in the epididymis [52]. In the brain, the administration of D-Asp also induced an increase in P levels [51].

### 3.2. In Vitro Experiments

Tomita et al. [56] examined the effect of D-Asp on the cultured testicular tissue of acrosome-green fluorescent protein-tagged (Acr-GFP) mice (5.5-day-postpartum pups). The flow cytometry analysis showed a dose-dependent decrease in the levels of Acr-GFP, an indicator of sperm differentiation in the testes of animals that were treated with D-Asp (Table 5). Moreover, immunohistochemical analysis using an anti-phospho-Histone H3 antibody evaluating the effect of D-Asp on the mitotic activity of testicular tissue cultured for 14 days revealed the inhibition of mitosis that could be associated with the presence of 10 mM D-Asp. Similarly, when an antibody was used against Synaptonemal Complex Protein 1 (SYCP-1), a meiotic protein marker, the authors observed a reduction in the number of meiotic cells in the testicular tissue slides that were treated with 10 mM D-Asp for 21 days. These results indicate that exogenous D-Asp suppresses the differentiation of germ cells in mouse testis by a mechanism that is yet to be understood.

Conflicting results were obtained using the GC-1 cell lines derived from immortalized type-B mouse spermatogonia that retained the markers of mitotic germ cells [57]. The incubation of these cells in a culture medium supplemented with 200 µM D-Asp or 50 µM NMDA resulted in higher expression of phospho-Extracellular signal-regulated kinases 1/2 (P-ERK1/2), phospho-protein-kinase B (P-AKT), PCNA, and Aurora B, demonstrating the direct effect of D-Asp and NMDA on the mitotic activity of spermatogonia (Table 5). In D-Asp-treated GC-1 cells, higher levels of P450 aro (mRNA and protein) and ERβ were also found. Furthermore, the expression of the main AMPAR subunits GluA1 and GluA2/3 has been recently described in spermatogonial GC-1 cells. GC-1 cells showed significantly higher levels of expression of both GluA1 and GluA2/3 after incubation with D-Asp or NMDA [14].

A recent study on murine Leydig cells demonstrated that the treatment with D-Asp alone did not induce any notable variation in the release of T or expression of LH receptor (LHR) protein [58]. On the other hand, the addition of human chorionic gonadotropin (hCG) significantly increased the T levels and expression of the StAR protein gene (Table 5) [58].

In vitro treatment of prepubertal rat testes (homogenates, explants, and cell suspensions) with 0–1 mM D-Asp enhanced lipid peroxidation, as confirmed by the increased production of cytosolic and mitochondrial ROS and MDA [59] (Table 5). This oxidative stress, associated with D-Asp exposure, was abrogated following the supplementation of L-arginine to the medium.

The direct endocrine role of D-Asp was validated by using both cultured immature (ILCs obtained from 35-day-old rats) [50] and mature [60,61] Leydig cells. After 12 h following the in vitro supplementation of 0.2 mM D-Asp, the ILCs showed a rapid uptake of D-Asp, and the levels of StAR protein increased significantly [50] (Table 5). At the molecular level, a significant increase in the levels of StAR, P450scc, and 3β-HSD mRNAs was found at 2, 4, and 12 h after D-Asp administration, respectively [50]. After 24 h, D-Asp administration was associated with a significant increase in the synthesis of androstenedione and T (Table 5).

Nagata et al. [60,61] described the time-dependent increase in T synthesis following D-Asp administration in mature rat Leydig cells. The purified Leydig cells were cultured for different time periods in the presence or absence (control) of D-Asp. An increased production of T was strongly correlated with the amount of D-Asp that was incorporated into the Leydig cells (Table 5). The authors demonstrated that D-Asp upregulated the production of T in the Leydig cells by enhancing the expression of StAR [61]. After incubation with D-Asp, the purified rat Leydig cells were incubated in a medium containing 5 mIU/mL of hCG for 2 h. It was observed that D-Asp increased the hCG-induced T production in these cells. In the presence of D-Asp alone, T production was increased only after exposure to D-Asp for more than 3 h [61].

Topo et al. [50], in accordance with these findings, demonstrated that the addition of 0.1 or 1.0 mM Na-D-Asp in a culture medium containing purified rat Leydig cells for 60 min caused an increase in both T and cAMP levels (Table 5).

Furthermore, to confirm the direct effect of D-Asp on neurosteroidogenic enzyme activities, homogenates of rat brain were incubated with different substrates (cholesterol, P, or T), with or without the addition of D-Asp [51]. Enzymatic activities were measured by evaluating the rates of in vitro conversion of (i) cholesterol to P, T, and E2; (ii) P to T and E2; and (iii) T to E2 (data not shown). These experiments collectively demonstrated that the addition of D-Asp to brain homogenate in the presence of a substrate induces a significant increase in the levels of P, T, and E2, suggesting that this amino acid upregulates the local activity of steroidogenic enzymes.

In vitro experiments conducted on isolated adenohypophysis or adenohypophysis co-incubated with hypothalamus have shown that the release of LH is caused by the direct action of D-Asp on the pituitary gland and mediated by the indirect action of NMDA on the hypothalamus (Table 5) [41,42]. This was because D-Asp also induced the release of Gonadotropin Releasing Hormone (GnRH) from the hypothalamus, which in turn was directly responsible for the D-Asp-induced LH secretion from the pituitary gland [41]. Topo et al. [50] demonstrated in rat models that pituitary D-Asp facilitated the synthesis and release of LH by involving cyclic guanosine monophosphate (cGMP) as the second messenger (Table 5).

Finally, D-Asp stimulated the secretion of PRL, which was inhibited by an NMDA receptor antagonist [63]. When anterior pituitary cells were cultured in the presence of posterior pituitary cells, NMDA did not modify the release of PRL or gamma-aminobutyric acid (GABA), while D-Asp increased the secretion of PRL and decreased the release of GABA (Table 5) [64].

D-Asp significantly increased the release of oxytocin from the hypothalamus and decreased the release of oxytocin from the posterior pituitary (Table 5) [62]. R-2-amino-5-phosphonopentanoate (AP-5) (a specific NMDA receptor antagonist) reduced the effect of D-Asp in the hypothalamus but not in the posterior pituitary. The activation of non-NMDA receptors and group-I mGluRs stimulated the release of oxytocin from hypothalamic nuclei, whereas NMDA inhibited the oxytocinergic terminals in the posterior pituitary. The same authors [63] also demonstrated that D-Asp stimulated the release of a luteinizing hormone-releasing hormone (LHRH), alpha-melanocyte-stimulating hormone (alpha-MSH), and GABA, and inhibited the release of dopamine in the hypothalamus, through the interaction with NMDA receptors. D-Asp increased the activity of nitric oxide synthase (NOS), and its effects on the release of LHRH and hypothalamic GABA were reduced by inhibiting NOS. In the posterior pituitary gland, D-Asp inhibited the release of GABA but had no effect on dopamine or alpha-MSH.

In the rat, certain sexual responses, such as penile erection, are controlled by neural circuits in the brain and spine, which are stimulated by the binding of excitatory amino acids (EAAs) to the postsynaptic NMDAR. In the hypothalamus, the EAA/NMDAR interaction triggers the activation of neuronal nitric oxide synthase (nNOS) to produce nitric oxide (NO). The local synthesis of this neurotransmitter in the penile nerve terminals results in the relaxation of the corpora cavernosa and erection. It is assumed that, during sexual activity, NO participates in the ejaculation, and inhibits voiding reflexes in the bladder. In vitro studies have demonstrated that all the essential NMDAR subunits are present in the lower urogenital tract, the tissues bind to an NMDAR ligand, and the NMDAR antagonists induce the relaxation of tissue strips [65].

In summary, several in vivo and in vitro studies carried out on mouse and rat models have contributed in clarifying the molecular pathways that are activated by D-Asp in the synthesis/release of hormones and gametogenesis. These studies essentially focused on the effects of D-Asp on the biosynthesis of T in males. The studies on the role of D-Asp in the female reproductive processes are rare. The repeated administration of D-Asp in sexually immature mice and sexually mature rats has shown that D-Asp induces an increase in the secretion of LH and T, suggesting indirect involvement of D-Asp in T synthesis, through the hypothalamus–pituitary–testis axis. In vitro experiments have demonstrated that D-Asp also has a direct effect on the pituitary and Leydig cells, as well as on spermatogonial proliferation. Some studies have suggested that excessive accumulation of D-Asp can activate mechanisms of oxidative stress in the testes [49], although no information is available on follow-up.

## 4. Livestock Animals

The presence and accumulation of D-Asp in endocrine and neuroendocrine tissues, and its consequent effects, have led the scientific community to investigate whether these effects are also present in livestock animals and humans. Thus, new opportunities have emerged regarding the use of this amino acid for therapeutic purposes, as well as in the management of livestock reproduction for productive purposes. For these reasons, attempts have been made to evaluate the affinity of this amino acid to certain endocrine glands and gonads by verifying the natural content, and therefore the ability of these tissues to take up D-Asp following its administration. Hence, from a physiological point of view, it is relevant to study the mechanisms that are induced by the administration of this amino acid.

Given the widespread presence of this amino acid in several plant species, its natural occurrence could be the diet [6]. The particularly high concentration of D-Asp in certain foods could provide novel methods to condition the reproductive activity in livestock animals. This possibility has already been investigated for certain fodders, which have been used over time to induce the so-called “food flushing” [66]. Accordingly, under normal dietary conditions, the concentration of D-Asp in the plasma is low; following the dietary consumption of foods that are particularly rich in D-Asp, its concentration may increase in the blood and many organs, including the brain and endocrine glands.

In sheep (*Ovis aries*), the highest tissue concentration of D-Asp was observed in the pineal gland, whereas the pituitary gland had the highest capability to store D-Asp [67]. The concentration of D-Asp in the sheep ovary was approximately 260 nM/g of tissue, whereas in the testis of pig, it was 22–40 nM/g tissue [68,69,70,71]. However, this value may be significantly regulated by the tissue content of D-AspO [72,73], as demonstrated by using targeted deletion of the D-AspO gene (DDO^-/-^) in mice [9,74,75].

### 4.1. In Vivo Experiments

In livestock, very few studies are available on D-Asp treatment, whereas a relatively higher number of studies have employed compounds such as DL aspartic acid (DL-Asp) or N-methyl DL-aspartic acid (NMA). These last studies, however, do not allow for the discrimination between the effects related to D- and L-Asp, and for this reason, they were not reported in the tables of this review but were discussed in the text.

Results obtained by in vivo long-term experiments on D-Asp administrations are reported in Table 6a where it is noteworthy that a detailed analysis of the effects of long-term D-Asp administration on the transcriptomic activity of enzymes and receptors involved in reproductive activity was conducted on roosters of broiler chicken (*Gallus gallus domesticus*) (Table 6a). Male broiler breeders (55 weeks old) were administered four different doses (0, 100, 200, or 300 mg/kg b.w.) of D-Asp for 12 weeks [76]. The doses of 100–200 mg/kg were the most effective in causing an increase in the transcript levels of StAR, P450scc, AR, LHR, 3β-HSD, PCNA, glutamate ionotropic receptor NMDA type subunit 1 (GRIN1), and glutamate ionotropic receptor NMDA type subunit 2B (GRIN2B) in the testis (Table 6a). Furthermore, D-Asp improved the quality and fertility of fresh and post-thawed sperm. Particularly, the oral administration of encapsulated 200 mg/die D-Asp improved the total and progressive sperm motility and plasma membrane integrity. In post-thawed conditions, D-Asp increased the total and progressive sperm motility, fertility, and hatchability, as well as plasma membrane integrity and mitochondrial activity. However, the percentages of live, early apoptotic, and dead spermatozoa were not significantly affected by D-Asp treatment [77].

In ewes (*Ovis aries*), long-term administration of D-Asp induced a significant increase in the LH values (Table 6a) with respect to E2 or E2 + D-Asp treatments. Prolonged D-Asp treatment may be associated with time-dependent changes [67]. The frequency of LH pulses also tended to be lower in ewes that were infused with D-Asp. Hence, although short-term administration of NMDA appears to benefit reproductive activity, its prolonged exposure is associated with a decrease in gonadotropin secretion that possibly involves decreased GnRH secretion [78].

In male rabbits (*Oryctolagus cuniculus*) that were subjected to a biweekly semen collection schedule, an oral daily administration of D-L-Asp (1.3 gr/kg b.w.) for two weeks did not affect semen volume, but caused a significant increase in sperm concentration, as well as kinetics such as the percentage of motile spermatozoa, the path velocity, and the percentage of progressively motile spermatozoa [79]. The levels of L-Asp in blood serum and seminal plasma did not vary throughout the experimental period, whereas the concentration of D-Asp in the serum increased more than 4-fold (+433%) compared to the baseline levels at the end of the treatment, and was maintained at levels higher than baseline for up to three weeks after the end of the treatment. The concentration of D-Asp in the seminal plasma was higher than in blood serum before the start of the treatment. The levels of D-Asp significantly increased (+115%) following treatment and returned to baseline values within one week after the end of treatment [79].

Table 6b shows the findings of an in vivo short-term experiment carried out on sheep (*O. aries*). Within 24 h following the administration of D-Asp, its levels in the ovaries, brain, pituitary, and serum of sheep largely increased (Table 6b). The levels of NMDA in the pituitary, brain, and serum increased sharply within 12 h following D-Asp administration, reaching values that were three times higher than the baseline [67]. An increase in serum LH values was also described.

Several studies reported the effects of NMA administrations on pituitary hormone secretion. Preliminary studies on castrated rams revealed no effects of NMA administration on the concentration, pulse frequency, and amplitude of LH during the 4 h period following the first NMA injection [80]. However, a day later, mean LH concentrations were decreased in NMA-treated versus control individuals.

In barrows (*Suis suis*), the administration of NMA (2.5–5.0 mg/kg b.w.) induced an increase of GH (+883–1095%) [81]. In male NMA-treated boar (*S. suis*), the treatment with NMA (10 mg/kg b.w.) caused an increase in the concentrations of serum LH (+100%), GH (+117%), and T (+93%) [82].

In vivo studies on prepubertal gilts (*S. suis*) demonstrated that aspartate (50–150 mg/kg b.w.) induced a higher secretion of GH (+60–340%) compared to glutamate [83]. Since this effect was suppressed in gilts that were immunized against GHRF, the authors hypothesized that the modulation of GH secretion by these EAAs was mediated primarily at the hypothalamus level. Further GH secretion increased in cultured gilt adenohypophysis cells challenged with aspartate [83]. In prepubertal gilts, NMA administration (10 mg/kg b.w.) elevated serum levels of GH (+700%) and LH (+80%) [84]. In ovariectomized gilts, NMA administration (10 mg/kg b.w.) suppressed the secretion of LH (−33%) either in P- or placebo-treated individuals [85,86]. However, in gilts that were treated during the luteal phase of the oestrous cycle, NMA treatment increased the pulse frequency of LH (+125%) but decreased its mean concentration. On the other hand, in gilts that were treated during the follicular phase of the estrus cycle, LH secretion appeared to be unaffected. When GnRH was administered in NMA-treated gilts, serum LH concentrations in the follicular phase were lower during the 2 h period following GnRH administration compared to ovariectomized gilts, and intermediate in gilts in the luteal phase. In contrast, NMA induced the secretion of GH (+334%) and cortisol (+77%) irrespective of the reproductive status of the treated gilts [86]. An increase of PRL in ovariectomized NMA-treated gilts was also described [85].

In cycling mare (*Equus caballus*), the response to NMA (1 mg/kg b.w.) was dependent on the stage of the estrus cycle, and a significantly higher proportion of individuals in the luteal phase responded to NMA treatment compared to those in the follicular phase [87]. In anestrous and cycling mares, NMA suppressed the secretion of PRL, which would normally be observed during the non-breeding season. Therefore, differences in reproductive activity in mares during the non-breeding season are unlikely to reflect a change in the glutamatergic activity, and the effects of NMA on LH release depend on the stage of the estrus cycle and the circulating steroidal milieu [87].

In the same species, Sticker and colleagues [88], treating stallion, mare, and castrated horses with aspartate (2.85 mmol/kg b.w.) and NMA (1 mg/kg b.w.), observed a different treatment response with the prevalent release of GH with the former and LH- follicle-stimulating hormone (FSH) with the latter.

### 4.2. In Vitro Experiments

In boars, Lamanna and colleagues [68] demonstrated that the addition of D-Asp to the homogenates of testis was associated with an increase in T levels, whereas the addition of L-arginine (L-Arg) led to a T decrease. In addition, L-Arg completely inhibited the stimulating effects of D-Asp [69]. The putative involvement of D-Asp on the aromatase activity for the synthesis of estrogen in the testes was also evaluated. The aromatase activity was demonstrated using immunoreactivity of Leydig cells, and to a lesser extent, of germ cells. In vitro experiments have revealed that the addition of D-Asp to testicular tissue induced a significant increase in aromatase activity, as assessed by the conversion of T to E2. However, the maximum rate of enzymatic reaction was not influenced by D-Asp [68].

In bovine sperm, a treatment with a commercial mixture of Coenzyme Q10 (CoQ10), zinc, and D-Asp (CZA) counteracts the loss of sperm motility and an increase in sperm DNA fragmentation during its storage. Moreover, the use of CZA-treated sperm for in vitro fertilization significantly increased the efficiency of this technique in terms of blastocyst rate, and the obtained blastocysts contained a significantly lower percentage of apoptotic cells [89]. The treatment of sperm using CZA also negated the deleterious effects, such as DNA fragmentation, reduced fertilization, lower blastocyst rates, and quality, that could be caused by induced exogenous oxidative stress [90].

In another study that compared several sperm metabolism enhancer treatments, such as myo-inositol, pentoxifylline, penicillamine + hypotaurine + epinephrine mixture (PHE), caffeine, and CZA for protecting spermatozoa from oxidative damage during storage, it was observed that CZA was the most effective in terms of maintaining the total and progressive bovine sperm motility, as well as the curvilinear velocity, average path velocity, and amplitude of head displacement [91].

In summary, in livestock, D-Asp has been isolated from several organs and tissues that have shown to have a high storage capacity following its administration. While some information on the effects of D-Asp on the release of pituitary hormones is available, information on its effects on the biosynthesis of sex hormones is scarce. However, only few in vivo and in vitro experiments on D-Asp administration are available, whereas, in most of the cases, treatments were carried out by using NMA. Interestingly, recent studies on roosters demonstrated direct effects of D-Asp on steroidogenic enzymes, as well as on total and progressive sperm motility.

## 5. Primates

Although no papers are available on the effects of D-Asp administration in primates, few reports evaluated the effects of NMA treatment on the release of pituitary hormones. Wilson and Knobil [92] reported that NMA administration in adult female rhesus monkeys (*Macaca mulatta*) induced a large and rapid increase in plasma levels of LH, FSH, and PRL.

The intravenous bolus administration of increasing doses of NMA (1.5, 4.8, and 15.0 mg/kg b.w.) to orchiectomized rhesus monkeys, aged between 13 and 20 months, elicited a distinct discharge of LH 10–14 h after termination of the priming infusion of GnRH. The magnitude of LH was directly correlated with the amount of excitant injected. However, the administration of a higher dose of NMA (48 mg/kg b.w.) failed to induce a further LH release [93].

## 6. Humans

Despite the enormous therapeutic potential related to the effects of D-Asp administration, as highlighted in the various animal models used, the pharmacological use of this amino acids in human medicine is low and there is a little research. 

Following the administration of sodium D-Asp supplemented with vitamins, such as folic acid, B6 and B12 (Dadavit^®^) for 12 consecutive days, treated men showed a relevant increase of LH and T levels (Table 7) in a time-dependent manner [50]. Although, after six days of D-Asp administration, LH increased only 1.07-fold, after 12 days of treatment, the LH levels increased significantly. Three days after the suspension of D-Asp treatment, serum LH concentration nearly returned to baseline values. 

Considering a positive relationship of T and GH levels with mechanisms of muscular hypertrophy, such as increased protein synthesis and satellite cell proliferation, it was hypothesized that the pharmacological activity of D-Asp could be aimed at reducing fatigue during training and strengthening the muscle mass in athletes. However, trials on resistance-trained men demonstrated no changes in T levels or training outcomes after up to one month of D-Asp supplementation [94,97,98]. In contrast, an increase in the daily intake caused a reduction in T levels after 14 days of supplementation [94] (Table 7). The long-term (3-month) administration of D-Asp in a resistance-trained population was evaluated in a randomized controlled trial on healthy resistance-trained men. No changes in the levels of basal total or free T, together with a reduction in E2 levels, were observed after this treatment (Table 7). Hence, D-Asp supplementation was ineffective in changing T levels or positively affecting training outcomes in trained individuals [95].

Concentrations of D-Asp in men were related to the occurrence of hypo-fertile conditions. D’Aniello et al. [99] reported a higher concentration of D-Asp in seminal plasma and spermatozoa in normospermic individuals compared to oligoasthenoteratospermic individuals. This concentration was even lower in non-obstructive azoospermic donors. No other D-amino acids were observed at levels comparable to those of D-Asp in seminal plasma or spermatozoa [99]. Later, the same research team evaluated the impact of treatments with commercial products containing D-Asp (Dadavit^®^ or Genadis^®^) on sperm quality in sub-fertile patients and the rate of pregnancies that occurred with their partners [96]. After 90 days of D-Asp administration, the treated patients showed significantly increased sperm concentration and motility (Table 7). The sperm concentration increased 2-fold and 1.6-fold in oligoasthenozoospermic patients and asthenozoospermic patients, respectively. These positive effects were also observed for sperm motility. In addition, a significant increase was observed in the number of pregnancies in partners of the treated patients [96].

The pharmacological use of D-Asp, together with coenzyme Q10 and zinc, has been proposed as a treatment for male infertility problems [100]. In vitro studies demonstrated that the total and progressive sperm motility in both normo- and oligospermic samples did not vary following supplementation of CZA to the sperm medium followed by 6 h of incubation, whereas a significant decrease in these parameters was observed when parallel samples were incubated in the medium alone. CZA supplementation also prevented a decrease in sperm kinetics, especially in oligospermic samples. Moreover, treatment with CZA protected the spermatozoa from an increase in DNA fragmentation and lipid peroxidation [100]. However, this was a synergistic effect, since none of the components used individually at various concentrations was able to improve sperm performance compared to control. The beneficial effects of CZA treatment were confirmed in a further study [101], although the authors did not record any improvement in sperm quality related to DNA fragmentation following CZA supplementation.

In women, the levels of D-Asp in the follicular fluid were evaluated [102]. A negative relationship between D-Asp content in the follicular fluid and patient age was observed. On the other hand, the follicular concentration of D-Asp has been observed to be positively correlated to the percentage of good quality oocytes and the rate of fertilization [102].

The studies on humans confirm the findings of previous studies on other animal species, specifically, that the administration of D-Asp is associated with an increase in the levels of LH.

Further, a clear positive association was observed between D-Asp and T, leading to the development of pharmaceutical preparations containing D-Asp that were used to enhance muscle development and improve reproductive efficiency in cases of male infertility. In vitro studies revealed the promising effects of D-Asp in the improvement of sperm quality. Even in women, preliminary studies have indicated a positive association between D-Asp and fertility, emphasizing that the mechanism of action of D-Asp in the control of reproductive activity has common effects that are detectable in both genders.

## 7. Conclusions and Future Perspectives

**Figure 1:**
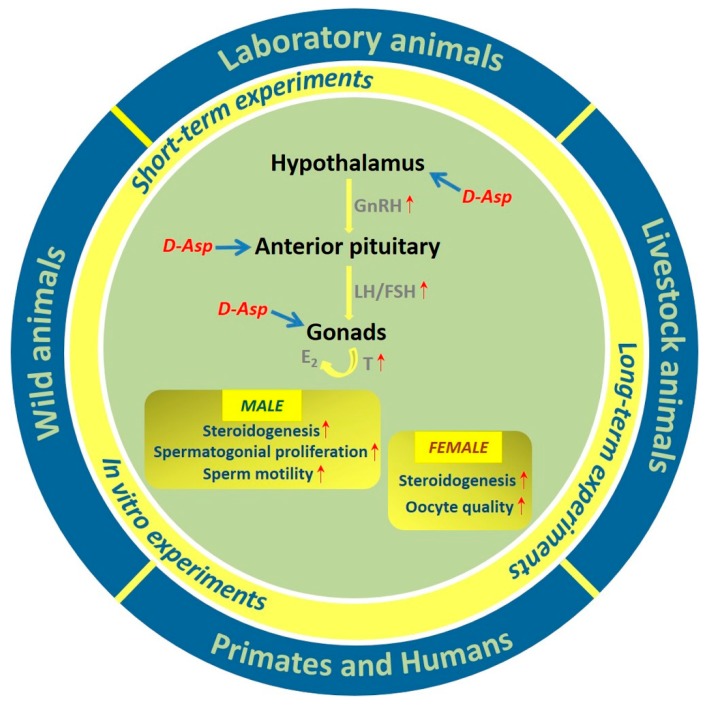
The scheme summarizes the effects of D-Asp administrations on the hypothalamic–pituitary–gonad axis in different classes of vertebrates. The effects of the amino acid on the male and female reproductive process are shown in the boxes.

This paper gathers information on the animal models and experimental designs that have allowed researchers to demonstrate the prominent role played by D-Asp and its methylated form, NMDA, in the reproduction of vertebrates, as schematically showed in Figure 1. A wealth of information is provided on the mechanisms and times of action of this amino acid, as well as the tissue concentrations necessary for optimum function. The aim of the review was to provide critical evaluations of the experimental designs and the obtained results to readers in order to facilitate the formulation of new research projects with novel applications.

The hypothalamus–hypophysis–gonad axis is a likely candidate to be the target of D-Asp and NMDA as it contains the highest tissue levels of D-Asp, has the capacity to accumulate this amino acid, and responds following its administration. In addition, the gonads possess enzymatic systems for the biosynthesis and degradation of D-Asp.

The majority of results from in vivo and in vitro experiments on different vertebrate species suggest that D-Asp acts as an excitatory molecule that induces the release of hormones (LH, PRL, and GH) by the anterior pituitary, and simultaneously directly induces the biosynthesis and release of T by stimulating the protein expression of StAR in Leydig cells. Furthermore, D-Asp is involved in the synthesis of DHT and E2 through the activation of 5αRed and P450 aro, respectively.

D-Asp plays a physiological role when administered in either a single dose (parenteral administration) or for long periods (intraperitoneal or oral administration). When this amino acid is administrated chronically, it is accumulated at a lower rate in the gonads, possibly due to the activation of the D-AspO. The in vivo or in vitro short-term experiments suggest that this amino acid is able to rapidly increase the gene and protein expressions of enzymes involved in steroidogenesis. In immature germ cells, D-Asp quickly activates the pathways of cell proliferation, directly influencing spermatogenesis. The effects of this amino acid, when administered in a single dose, diminish after 24 h.

Experiments have commonly been performed on frog, lizard, mouse, and rat, whereas studies on livestock animals and humans are scarce. The experiments conducted on seasonally reproductive species suggest that D-Asp contributes to the switching on/off of the androgenic or estrogenic pathways during reproductive and non-reproductive periods. 

Wistar rat has been by far the most commonly used experimental model for these studies. In vivo and in vitro experiments carried out on this species have demonstrated that D-Asp acts at all levels of the hypothalamic–pituitary–gonadal axis: (1) it can affect the secretion of GnRH; (2) regulates the secretion of pituitary hormones; and (3) exerts a local effect on gonads, inducing the secretion of sex steroid hormones.

The experiments carried out on isolated Leydig cells and GC-1 spermatogonia demonstrated a direct effect of D-Asp on the steroidogenic pathway and spermatogenesis, respectively. In Leydig cells, D-Asp enhances the gene and protein expression of enzymes involved in the steroidogenic cascade with consequent T release, whereas, in spermatogonia, it activates the proliferative pathway. 

Attempts using D-Asp to improve the reproductive activity of animals of commercial interest have yielded mixed results. The increased transcriptomic activity of enzymes and receptors involved in the reproductive activity in D-Asp-treated broiler roosters suggests a higher complexity of the mechanism of action of D-Asp on the reproductive processes.

The close relationship between D-Asp and reproductive activity has been particularly explained by the effects exerted by this amino acid on semen quality, suggesting further potential applications in the field of andrology and medically-assisted procreation techniques. Microinjection of NMDA into PVN has been demonstrated to reduce the latency of intromission and facilitate ejaculation during copulation in rat models. Preliminary approaches to this topic have yielded promising results, and the potential of D-Asp is expected to be the subject for future in-depth investigations.

Although the greatest efforts have so far been focused on the function of D-Asp in reproduction, numerous questions remain unanswered. First, the duration of the effect of D-Asp, after the suspension of its treatment, is not clear. Second, it needs to be answered whether the accumulation of D-Asp can activate mechanisms of oxidative stress in the tissues. Third, the role of D-Asp in female reproduction is not fully understood.

Finally, different research groups need to work together to explore the potential role of D-Asp in mediating functions of the central nervous system. Preliminary studies have suggested that neurosteroid biosynthesis is a possible target for neuronal D-Asp. Since it has been shown that neurosteroids, and particularly estrogen, exert a protective effect on nervous cells, as well as play a key role in mechanisms modulating the reproductive behavior, further research in this field is open.

In conclusion, several studies are still required to understand the potential of D-Asp in tackling human infertility, as well as in livestock reproduction. Undoubtedly, the numerous experiments conducted in laboratories will be of immense help to researchers working in this field.

## Figures and Tables

**Table 1:** Wild animals: in vivo experiments.

	BREEDING SEASON				OUT-OF-BREEDING SEASON				
**a** **) Long-term experiments**									
Injections of 2 µmol/g b.w./d D-Asp for 10–15 days	**D-Asp uptake**		**Increase/Decrease**		**D-Asp uptake**		**Increase/Decrease**		**References**
***P. esculentus*** *♂*									
*Testis*	+96%		>1000% E_2_ +19% StAR mRNA +80% P450 aro mRNA		+100%		+67% T −90% E_2_ +500% [D-AspO] +43% StAR mRNA +54% 5α-Red2 mRNA		Burrone et al. [33]
*Brain*	+450%		−60% T +100% E_2_ +43% P-CREB +40% P450 aro mRNA +175% P450 aro protein −25 % AR mRNA +25% ERα mRNA		+400%		+140% D-AspO activity +120% caspase 3		Burrone et al. [36] Burrone et al. [33] Santillo et al. [35]
**b) Short-term experiments**									
One Injection of 2 µmol/g b.w./ D-Asp	**D-Asp uptake**		**Increase/Decrease**		**D-Asp uptake**		**Increase/Decrease**		**References**
	***3 h***	***6 h***	***3 h***	***6 h***	***3 h***	***6 h***	***3 h***	***6 h***	
***P. esculentus*** *♂*									
*Testis*					+500%	+800%	+475% T	+25% T	Raucci et al. [40]
*Serum*							+255% T	+33% T	Raucci and Di Fiore [29]
♀									
*Ovary*					+900%	+775%	−52% T	−21% T	Di Fiore et al. [31]
*Serum*			−82% T	−47% T			−50% T	−83% T	Raucci and Di Fiore [29]
***P. s. sicula*** *♂*									
*Testis*	+140%	+100%	+32% T −50% E_2_	+20% T −37% E_2_	+800%	+500%	+96% T −71% E_2_ +23% c-Kit −50% PK	+78% T −42% E_2_ +130% PTK +29% PCNA	Raucci et al. [27] Raucci and Di Fiore [28]
*Serum*			+5% T −25% E_2_	+23% T −75% E_2_			+450% T −61% E_2_	+1150% T −84% E_2_	
♀									
*Ovary*					>1000%	>1000%	−37% T +200% E_2_	−12% T +80% E_2_	Assisi et al. [30]
*Serum*							−42% T +300% E_2_	−28% T +100% E_2_	Raucci and Di Fiore [32]

**Table 2:** Wild animals: in vitro experiments.

	Incubation	Increase/Decrease	References
***P. esculentus*** (breeding season)			
*Ovarian follicles*	+2 μmol/mL D-Asp (3 h)	−62%T	Di Fiore et al. [31]
*Ovarian follicles + pituitary*	+2 μmol/mL D-Asp (3 h)	−56%T	
***P. s. sicula*** (out-of-breeding season)			
*Ovarian tissue*	+2 μmol/mL D-Asp (3 h)	+700% Aromatase activity	Assisi et al. [30]
*Acetonic powder of ovarian follicles*	+2 μmol/mL D-Asp (3 h)	+566% Aromatase activity	
***A. platyrhyncos*** (out-of-breeding season)			
*Testis slices*	+1–2 mM D-Asp (3 h)	+33–150% T	Di Fiore et al. [25]

**Table 3:** Laboratory animals: in vivo long-term experiments.

	D-Asp Uptake	Increase/Decrease	References
***Sexually immature B6N Mice***		+14–20% IVF	Raspa et al. [48]
20 mM D-Asp drinking solution for 1–6 weeks			
*♂*			
*Testis*		+71% T +300–360% Epitestosterone +170% LH	
*Serum*		+25–46% T +36–81% Epitestosterone +36–46% LH	
***Wistar Rats*** ***Prepubertal*** Injections of 100–500 mg/kg b.w./d D-Asp for 7 days			
*♂*			
*Testis*		+ 75% mitochondrial ROS +30% cytosol ROS +20–50% MDA, hydroperoxide levels, LPO, GSH, catalase activity, GPX, GST, LDH, 3β-HSD, NO, D-AspO −15% MDH	Chandrashakar and Muralidhara [49]
***Sexually mature*** 20 mM D-Asp drinking solution			
*♂*			
*Testis* (treatment for 12–15 days)	+460–720%	+70% T +80% Androstenedione +100–120% P-ERK1/2 +37% StAR (mRNA-protein) +33% P450scc mRNA +38% 3β-HSD mRNA −25% P450 aro protein +55% AR protein −47% ERα protein +130% NR1-NR2A mRNAs	Topo et al. [50] Raucci et al. [40] Santillo et al. [13]
*Pituitary* (treatment for 12 days)	+580%		Topo et al. [50]
*Serum* (treatment for 12–15 days)	+100%	+100–120% T +51–128% LH +40% Androstenedione	Topo et al. [50] Raucci et al. [40]
*Brain* (treatment for 30 days)	+100%	+40% P +110% T +35% E_2_	Di Fiore et al. [51]

**Table 4:** Laboratory animals: in vivo short-term experiments.

	D-Asp Uptake			Increase/Decrease			References
	***30 min***	***1*** **–*2 h***	***5*** **–*8 h***	***30 min***	***1*** **–*2 h***	***5*** **–*8 h***	
***Wistar Rats*** One injection of 2 µmol/g b.w. D-Asp							
*♂*							
*Testis*		+133%	+311%		+150% NMDA		D’Aniello et al. [41,42]
*Adenohypophysi* *s*		>1000%	>1000%		+125–166% NMDA		
*Hypothalamu* *s*		+152%	+267%		+200–860% NMDA		
*Epididymis*	+500%	+110%	+70%	+ 900% T +62% DHT +100% E_2_ +111 % AR mRNA +233% P450 aro mRNA +166% ERα mRNA	+600% T +62% DHT +80% E_2_ +200 % 5αRed1 mRNA +150% P450 aro mRNA +366% ERα mRNA	+ 450% T +112% DHT +74% E_2_ +466 % 5αRed1 mRNA +78% % 5αRed2 mRNA +100% P450 aro mRNA +166% ERα	Falvo et al. [52]
*Serum*		>1000%	>1000%	+145% PRL	+36% T +34% P +110% LH +200% PRL +97% GH	+236% T +172% P +145% LH +161% GH	D’Aniello et al. [41,42]
*Brain*	+288%	+288%	+211%	+26% T +28% E_2_	+80% P +66% T +42% E_2_	+90% P +93% T +85% E_2_	Di Fiore et al. [51]

**Table 5:** Laboratory animals: in vitro experiments.

Incubation		Increase/Decrease	References
***Mouse***			
*Cultured ICR testis tissue*	+1–10 mM D-Asp (4 wks)	−21–71% Acr-GFP	Tomita et al [56]
*GC-1 spermatogonia*	+200 µM D-Asp (0.5-2 h)	+60–100% GluA1 +157% GluA2/3 +83% P-ERK1/2 +183% P-Akt +29% PCNA +84% AuroraB +266% P450 aro mRNA +62% P450 aro protein +112% ERβ	Santillo et al. [14,57]
	+50 µM NMDA (0.5–4 h)	+66–116% GluA1 +63–75% GluA2/3 +66–133% P-ERK1/2 +33–133% P-ERK2 +40–50% PCNA +40% AuroraB	Santillo et al. [14]
*Leydig cells*	+0.1 nM D-Asp+10 ng/mL hCG (48 h)	+25% T + 83% StAR protein	Di Nisio et al. [58]
***Wistar Rat***			
*Prepubertal testis*	+0–1 mM D-Asp (2 h)	+50–280% MDA (cytosol) +83–183% MDA (mitochondria) +143% ROS +332% LPO +82% HP	Chandrashakar and Muralidhara [49,59]
*Immature Leydig cells*	+0.2 mM D-Asp (2–24 h)	+112% StAR mRNA +14% P450scc mRNA +33% 3β-HSD mRNA +36% StAR protein +100% Androstenedione release +185% T release	Raucci et al. [40]
*Mature Leydig cells*	+200 µM D-Asp (16 h) +200 µM D-Asp (16 h)+ 5 mIU hCG/ml (2 h)	+50% T +250% StAR mRNA +90% StAR protein	Nagata et al. [60,61]
	+0.1 or 1.0 mM D-Asp (1h)	+142–200% T +200–325% cAMP	Topo et al. [50]
*Hypothalamus*	+1 mM D-Asp (0.5 h)	+105% oxytocin	Pampillo et al. [62]
*Adenohypophysis*	+0.1–1 mM D-Asp (1 h)	+150–300% PRL +16% LH +166% GH + 150–200% cGMP	D’Aniello et al. [41,42] Topo et al. [50]
	+0.1 mM NMDA (1 h)	+185% GH +83% LH	
	+0.1–1 mM D-Asp (4 h) (anterior and posterior hypophysis cell coltures)	+11–13% PRL	Pampillo et al. [63]
	+0.1–1 mM D-Asp (4 h) (anterior hypophysis cell coltures)	+15–25 % PRL	
	+ 0.01–0.1 mM NMDA (4 h) (anterior hypophysis cell coltures)	+71–110 % PRL	
*Neurohypophysis*	+0.1 mM D-Asp (0.5 h) +0.1 mM NMDA (0.5 h)	−35% oxytocin −45% oxytocin	Pampillo et al. [62]
*Adenohypophysis+* *hypothalamus*	+1 mM D-Asp (0.5–4 h)	+450% PRL +72% LH +33% GH	D’Aniello et al. [41,42]
	+0.1 mM NMDA (1 h)	+202% LH +87% GH	

**Table 6:** Livestock animals: in vivo experiments.

**a) Long-term experiments**	**D-Asp uptake**	**Increase/Decrease**	**References**
***G. g. domesticus***			
Oral administration of 100–200 mg D-Asp/kg for 12 wks			
*Testis*		+120–680% StAR mRNA +100–1000% P450scc mRNA +100–337% 3β-HSD mRNA +100–766% AR mRNA +120% LHR mRNA +100% Grin1 mRNA +300–1150% Grin2b mRNA +250–1800% PCNA mRNA	Ansari et al. [76]
*Serum*		+10–24% T	
*Sperm*		+sperm motility +Fertility +Hatchability +Plasma membrane integrity +Mitochondrial activity	Ansari et al. [77]
***O. aries***			
S.C. administration of 22.2 mg D-Asp/kg b.w. every 3 or 6 days for 1 month			
*Serum*		+40% LH	Boni et al. [67]
**b) Short-term experiments**	**D-Asp uptake**	**Increase/Decrease**	**References**
***O. aries***			
S.C. injection of 44.4 mg D-Asp/kg b.w.			
*Ovary*	+650% (24 h)		Boni et al. [67]
*Pituitary*	>1000% (12–24 h)	+200% NMDA (12 h)	
*Brain*	+125% (12–24 h)	+250% NMDA (12 h)	
*Serum*	>1000% (3 h)	>1000% NMDA (5–12 h) +40% LH (2 h)	

**Table 7:** Humans: in vivo long-term experiments.

	Increase/Decrease	References
Oral administration of:		
DADAVit^®^ for 12 days	+33% LH +42% T	Topo et al. [50]
6 g D-Asp for 14 days	−15% T in resistance trained men	Melville et al. [94]
6 g D-Asp for 3 months	−95% E_2_ in resistance trained men No positively affecting training outcomes	Melville et al. [95]
DADAVit^®^ or GENADIS^®^ for 3 months	+sperm concentration and motility in asthenozoospermic and oligoasthenozoospermic men +pregnancy rate	D’Aniello et al. [96]
